# Tuning Into Affect and Appetite in Caregivers, and Its Association With Recognising and Responding to Infant Appetite Cues

**DOI:** 10.1111/mcn.70099

**Published:** 2025-08-31

**Authors:** Shihui Yu, Alison Fildes, Pam Birtill, Tang Tang, Marion M. Hetherington

**Affiliations:** ^1^ School of Psychology University of Leeds Leeds UK; ^2^ School of Design University of Leeds Leeds UK

**Keywords:** affect, alexithymia, appetite, eating traits, infants, responsive feeding

## Abstract

Positive mealtime interactions shape infant eating patterns potentially promoting appetite regulation. This study investigated whether caregivers who “tune‐in” to their own internal affect and appetite cues, can also recognise and respond to their infant's appetite cues via responsive feeding (RF). Caregivers (*N* = 445; mean age: 33.5 ± 4.7 years) with children aged 5–28 m participated in an online survey in August 2023. Caregivers' RF practices, mealtime emotions, eating traits, alexithymia (impaired capacity to identify and express emotions) and their infant's eating traits were administered using validated questionnaires. Recent mealtime experiences were described through an open‐ended question. Caregivers who relied on interoceptive cues in eating scored high on recognising infant appetite cues (*R* ^2^ = 0.11, *F*(1, 396) = 5.40, *p* < 0.001). Whereas caregivers with alexithymia reported poorer ability to recognise infant appetite cues (*R* ^2^ = 0.12, *F*(7, 399) = 7.53, *p* < 0.001) and less positive mealtime emotions (*R* ^2^ = 0.12, *F*(7, 399) = 7.49, *p* < 0.001) compared to those without alexithymia. Caregivers' capacity to “tune‐in” to their own internal satiation cues inversely mediated the relationship between caregivers' alexithymia and their recognition of infant mealtime appetite cues. Infant eating traits (Food Responsiveness and Satiety Responsiveness) were associated with parental use of food to calm. Overall, RF was associated with mealtime emotions, parental ability to “tune‐in” to their own affect (alexithymia) and appetite, and child's appetitive traits. Developing caregiver's awareness and responsiveness to their own and their child's affect and appetite cues may promote RF practices.

## Summary

- Caregivers who scored high on alexithymia might struggle with recognising their own, and their infants' hunger and satiety cues, leading to fewer responsive feeding (RF) practices and less positive mealtime experiences.
- Caregivers who were more in tune with their own hunger cues were better at recognising their infant's hunger and satiety signals during feeding.
- Future research should explore barriers for caregivers with alexithymia in adopting RF practices and ways to support them in fostering healthy eating habits in children.

## 1 Introduction

Infant mealtimes involve the provision of nutritious foods and the opportunity for bidirectional communication between the caregiver and the child. Positive mealtime interactions relate to a number of environmental or social elements, such as mealtime settings, positioning, child participation or engagement, mealtime distractions, verbal communication during mealtimes, food provided, and parental responsiveness to child appetitive cues (Shloim et al. 2015). It is demonstrated that infants are increasingly capable of communicating sophisticated appetitive cues, such as hunger and satiation (Hetherington 2017). Being able to identify and then respond to a child's appetitive cues is important since positive mealtime interactions may influence which foods are eaten, how much the child eats, and the development of eating patterns (Daniels et al. 2009; Lakshman et al. 2015; Paul et al. 2014; Van der Veek et al. 2019). In a longitudinal observational study, mothers who reported greater awareness of, and sensitivity to, infants' mental states (thoughts, feelings, and desires), were more responsive and involved during feeding, than mothers who were less sensitive (Farrow and Blissett 2014).

Parental sensitivity to infant cues may depend on the extent to which parents are able to recognise and respond to their own internal cues including affect and appetite. This ability varies in caregivers, and in some there is a reduced capacity to identify and express emotions and bodily sensations, known as alexithymia (Taylor 1984). In clinical studies, patients with alexithymia showed an apparent inability to verbalise feelings, a reality‐based concrete cognitive style, and impoverished inner emotional and fantasy lives (Taylor et al. 1991; Taylor et al. 1985). Previous research suggested that alexithymia is substantially a communicative disorder, which has been studied through the content analysis of speech, with alexithymic patients reported to use limited emotional vocabulary (Taylor 1984). In a study that followed a structured clinical procedure and included free play observation, high maternal alexithymia was associated with higher depression and lower mother–infant relationship quality compared to low maternal alexithymia (Yürümez et al. 2014). Evidence from the FinnBrain Cohort study indicated that high maternal alexithymia scores were related to weak maternal sensitivity, and poor negative emotion regulation which were measured through video‐recorded mother–infant free play interaction in a laboratory setting (Ahrnberg et al. 2021).

In the appetite domain, The Trust Model originally proposed by Satter (1986) emphasises the division of feeding responsibility between caregivers and children. Here responsive feeding (RF) practices that both recognise and respond promptly to a child's hunger and satiety cues, will promote their appetite regulation and may reduce obesity risk (Eneli et al. 2008). Parents who trust in their child's ability to “self‐regulate food intake by recognizing hunger, appetite, and satiety cues within the context of regular eating patterns” (Eneli et al. 2008, p. 2179), will enhance self‐regulation of appetite. In contrast, nonresponsive feeding may impair the expression of internal satiation cues overriding the ability to self‐regulate (Hurley et al. 2011).

Caregivers' feeding practices are influenced by both caregiver's characteristics such as body mass index (Shloim et al. 2015) and those of the child such as their weight status and appetitive traits (Moore et al. 2007; Webber et al. 2010). Twin studies demonstrate that parents feed their twin children differently depending on each child's eating behaviours (Farrow et al. 2009; Harris et al. 2016; Kininmonth et al. 2023). Additionally, caregivers adapt their feeding practices in response to their children's appetitive traits. For example, Miller et al. (2020) found that nonresponsive feeding practices, such as overt restriction and food reward, were adopted in response to children's avid appetite traits including Food Responsiveness and Emotional Overeating. Similarly, within the INSIGHT trial which assessed the sustained effects of a responsive parenting intervention, maternal feeding practices were modified by child eating traits (Ruggiero et al. 2021).

Eating traits have been aggregated and named “sensitivity to internal satiation cues”, which indicate an ability to “tune in” to internal appetite cues in adults (Chawner et al. 2022). This construct reflects a general capacity to recognise and respond to internal satiation cues involving eating mindfully, attending to changes in physical satisfaction and food appeal during satiation development (Chawner et al. 2022), and is shown to be associated with intuitive eating which refers to an awareness and trust of internal hunger and satiety cues used to determine when and how much to eat (Tylka and Kroon Van Diest 2013). The ability to recognise and respond to internal appetite cues may have implications for how mothers feed their infants and their motives to continue or to stop feeding in a single meal. Some mothers may encourage their child to exert control over food intake and they will stop feeding on noticing signs of disinterest or fullness (McNally et al. 2019). Similarly, parents may be aware that, for them, foods taste less pleasant as the meal progresses, and they may apply this to their infants by offering a variety of foods when disinterest in one food is observed (McNally et al. 2019).

To expand our understanding of how infant appetite cues are perceived, we previously investigated the role of individual differences. In response to video clips of infants demonstrating hunger and satiety cues, recognition was accurate but greater alexithymia scores were correlated with lower recognition of infant appetite cues (Yu et al. 2025). This online study indicated the need to explore alexithymia specifically in caregivers of young children, its association with RF practices and the experience of mealtimes at home, including affective tone during meals.

Therefore, the overall aim of the present investigation was to explore the extent to which caregivers' alexithymia, sensitivity towards their own satiation, in addition to their child's appetitive traits, predicted RF behaviours during mealtimes. Quantitative approaches were applied to investigate these dynamics, with a particular focus on integrating caregivers' lived experiences. There were four hypotheses (H1–H4):

Caregivers who score high on alexithymia will score low on self‐reported RF practices and report fewer positive mealtime emotions compared to those with low scores on alexithymia.

Caregivers who have high scores on RF practices will also report more positive experiences of mealtimes, with fewer challenges and distress.

Caregivers who have higher sensitivity to their own internal satiation cues, who score high on intuitive eating and reasons to stop eating which are more interoceptive than contextual, will have higher responsiveness to their child as measured by high scores for sensitivity to their child's hunger and satiety cues during feeding.

RF will be predicted by the caregiver's own ability to recognise and respond to (a) their own appetite cues, and (b) to their child's eating traits. Alexithymia will influence RF practices through reduced sensitivity to caregiver's own appetite cues.

To explore how caregivers who score high on alexithymia report their mealtime experiences and emotions, and whether their communication of experiences is different from caregivers without alexithymia, a qualitative component was included to investigate the emotional tone and relational dynamics of caregiver‐infant mealtime interactions, by analysing caregivers' reflections on their most recent feeding experiences.

## 2 Methods

### 2.1 Participants and Design

In this study, we aimed to recruit 500 participants via the Prolific platform (https://www.prolific.co/).

The sample size was determined based on the prevalence of alexithymia in the general population, where around 10% of people having problematically high alexithymia (Luminet et al. 2018). To enable a sufficient sample of caregivers with alexithymia, we planned to recruit approximately 500 caregivers to reach a sample of 50 with clinical threshold alexithymia.

The inclusion criteria were UK residents aged 18 and above, fluent in English, have at least one infant between 6 and 24 months old in the household. Caregivers with twins or multiple birth children were welcome to participate. Caregivers with full‐term infants with chronic, medical conditions which may affect feeding (e.g. neuro‐developmental disorders), or caregivers with preterm infants, or those who failed the engagement and attention checks were excluded from the study. Participants were compensated via Prolific for their time.

The study protocol was pre‐registered on the Open Science Framework (OSF): https://osf.io/5asn7/.

This study used both quantitative and qualitative data collection via Qualtrics (https://www.qualtrics.com/uk/). The study was advertised as “Exploring emotions and communication during mealtimes” with four sections which took approximately 12 min to complete. Section 1 consisted of general demographic questions including parenting status and child information. Section 2 consisted of measurements of caregiver and child appetite traits. Section 3 consisted of caregivers' self‐reported RF practices, and their emotional responses experienced during family mealtimes. Section 4 consisted of measurements of caregivers' response to satiation cues, intuitive eating behaviour, and alexithymia.

The survey concluded with an open‐ended question where caregivers were invited to provide detailed reflections on recent mealtime experiences, which could offer insights into the emotional and relational dynamics of feeding interactions. By capturing these narratives, the qualitative component enriched the study by complementing the quantitative findings, offering a deeper understanding of caregivers' lived experiences and the contextual factors that influence RF practices.

This study was approved by the University of Leeds School of Psychology Research Ethics Committee (Reference: PSCETHS‐680).

### 2.2 Procedure

**Figure 1:**
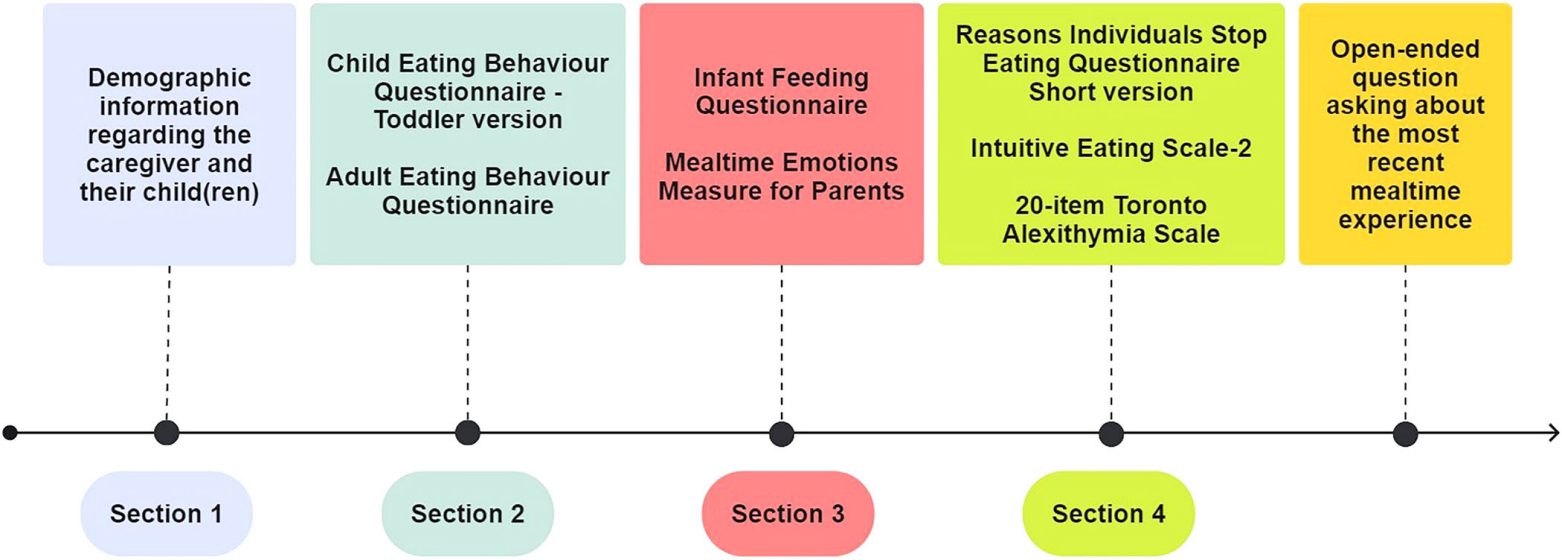
Timeline of study procedure following informed consent.

First, participants were informed about the study and invited to consent to participate (timeline is presented in Figure 1). Next participants completed the following: Section 1 asked general demographic questions about the caregiver and their child(ren) in their household. Participants were asked to refer to one specific infant within the target age group (6–24 months old) to complete following Section (5 min). For twins or triplets, caregivers were asked to select one of their children for the purpose of this study. Section 2 presented the Child Eating Behaviour Questionnaire for Toddlers, followed by the Adult Eating Behaviour Questionnaire (3 min). Section 3 contained the Infant Feeding Questionnaire and the Mealtime Emotions Measure for Parents (4 min). In Section 4, participants were asked to complete the Reasons Individuals Stop Eating Questionnaire short version, the Intuitive Eating Scale 2, as well as the 20‐item Toronto Alexithymia Scale (5 min). Questionnaire items were randomised within the sections.

This survey ended with an open‐ended question: “Thinking of the last mealtime you had with your child, can you describe at what time of the day you fed them, how did you feed them, and how did you feel about the mealtime interaction?” Participants who submitted their questionnaire were thanked, paid and offered an opportunity to register their interest in future studies via a separate contact form.

### 2.3 Measures

#### 2.3.1 Responsive Feeding Practices

To measure caregivers' self‐reported RF practices, the Infant Feeding Questionnaire (IFQ) (Baughcum et al. 2001) was used. It is a tool with 20 items evaluating early feeding practices or beliefs that might lead to childhood obesity, originally used with infants aged between 11 and 23 months. It contains seven constructs such as Awareness of Infant's Hunger and Satiety Cues, Feeding Infant on a Schedule, Using Food to Calm, Infant's Fussiness, Social Interaction during Feeding, in addition to constructs measuring caregivers' concerns about infant hunger and weight. Caregivers rated items on a 5‐point Likert scale, for example, “Did you worry that he was not eating enough?” from (0) Never to (4) Always. The rating scales for questions on feeding beliefs such as “He knew when he was hungry” were anchored from 0—disagree a lot to 4—agree a lot. Mean scores were calculated for each subscale.

#### 2.3.2 Caregivers' Emotions in Typical Family Mealtimes

To capture how caregivers emotionally experience mealtimes with their family, the Mealtime Emotions Measure for Parents (MEM‐P) (White et al. 2022) was used. MEM‐P is a self‐report tool with three constructs: Anxiety; Stress and Anger; and Efficacy to examine caregivers' emotional responses experienced during family mealtimes, adapted from the 13‐item Mealtime Emotions Measure for Adolescents (White et al. 2015). For the MEM‐P parents report frequency of recognising emotions, whether parents feel in control of their emotions, and their confidence in dealing with child distress. It contains 16 emotional responses on a 7‐point Likert Scale (1 = Never to 7 = Always).

#### 2.3.3 Alexithymia

To measure alexithymia, the 20‐item Toronto Alexithymia Scale (TAS‐20) (Bagby, Parker, et al. 1994; Bagby, Taylor, et al. 1994) was used. TAS‐20 is a self‐report measure of alexithymia containing three dimensions: Difficulty Identifying Feelings, Difficulty Describing Feelings, and Externally Oriented Thinking. It has 20 items and each of them is rated on a 5‐point Likert scale ranging from 1 (strongly disagree) to 5 (strongly agree). TAS‐20 uses cut‐off scoring with scores of 52–60 indicating potential alexithymia, and scores above 60 indicating present alexithymia. Therefore, the higher individual scores, the more alexithymia traits are present, and the more certainty there is that the response reaches the clinical threshold for alexithymia.

#### 2.3.4 Appetite Traits

To assess *children's eating behaviour traits*, five subscales from the Child Eating Behaviour Questionnaire for Toddlers (CEBQ‐T) (Herle et al. 2016) were included. The CEBQ‐T is a modified version especially for toddlers based on the validated and widely used Child Eating Behaviour Questionnaire (Wardle et al. 2001). The CEBQ‐T was originally developed with infants aged 18‐month‐olds. In this study, Food Responsiveness (FR, 4 items), Satiety Responsiveness (SR, 5 items), Slowness in Eating (SE, 4 items), Enjoyment of Food (EF, 4 items), and Food Fussiness (FF, 6 items) were included. In the CEBQ‐T, FR measures parental perceptions of infant's responsiveness to food cues and external eating; such as “My child's always asking for food.” SR measures items such as “My child gets full before his/her meal is finished.” SE consists of items that assess a child's speed of eating. EF, in addition to FR, reflects eating in response to environmental food cues. FF measures the tendency to be highly selective about which foods are eaten. A 5‐point Likert scale (1 = Never, 5 = Always) was used for caregivers to report how frequently their infant demonstrates these eating traits.

To measure *caregivers' appetite traits*, five subscales from the Adult Eating Behaviour Questionnaire (AEBQ) (Hunot et al. 2016), which are parallel to child appetitive traits measurements, named Food Responsiveness (FR, 4 items), Enjoyment of Food (EF, 4 items), Satiety Responsiveness (SR, 4 items), Slowness in Eating (SE, 4 items) and Food Fussiness (FF, 5 items) were included. SR measures individual's sensitivity to feelings of fullness. Poor SR is linked to overeating and overweight. Items are asked such as “I often get full before my meal is finished.” Whereas FR indicates the respondent's food approach and response to external food cues (“I often feel hungry when I am with someone who is eating”). Scores are anchored on a 5‐point Likert scale ranging from (1) strongly agree to (5) strongly disagree.

To measure *intuitive eating behaviours*, a subscale from the Intuitive Eating Scale‐2 (Tylka and Kroon Van Diest 2013) named Reliance on Hunger and Satiety Cues was used. It contains 6 questions, such as “I rely on my fullness (satiety) signals to tell me when to stop eating”, with scores to each item on a 5‐point Likert scale ranging from (1) strongly disagree to (5) strongly agree. The average score reflects individuals' trust in their internal hunger and satiety cues and reliance on these cues to guide their eating behaviours.

To measure an individual's *response to their own satiation cues*, the Reasons Individuals Stop Eating Questionnaire short version (RISE‐Q15) (Chawner et al. 2022) was included. It contains five subscales (Decreased Food Appeal, Physical Satisfaction, Planned Amount, Self‐Consciousness, and Decreased Priority of Eating) and each of them consists of 3 items, such as “my stomach is full”, “the food is no longer pleasant”, anchored on a 7‐point frequency scale ranging from (1) Never to (7) Always. The average score represents a general capacity to recognise the reasons for stopping eating via internal and contextual cues, whereas the DFA and PS indicate an ability to respond to interoceptive cues.

### 2.4 Data Analysis

#### 2.4.1 Quantitative Data

First, we characterised our sample by using descriptive data on demographics of caregiver and then child. The age, gender and feeding methods for each child were recorded. Milk feeding during the first 3 months of life was recorded with six options: “a. Breastfeeding only”, “b. Mostly breastfeeding, with some formula‐feeding”, “c. Equal amount of breastfeeding and formula‐feeding”, “d. Mostly formula‐feeding and some breastfeeding”, “e. Almost all formula‐feeding” and “f. Formula‐feeding only”. To help with the statistical analysis, responses were dichotomised into “Entirely or mostly breastfeeding” (a + b), or “Equally, entirely or mostly formula feeding” (c + d + e + f). Similarly, complementary feeding was reported from the following five options: “a. Exclusively spoon feeding”, “b. Mainly spoon feeding with some finger foods”, “c. A combination of spoon feeding and baby‐led weaning”, “d. Mainly baby‐led weaning with some spoon feeding”, “e. Exclusively baby‐led weaning”. Responses were again subsequently grouped as “Exclusively or mostly spoon feeding” (a + b), “A combination of spoon feeding and baby‐led weaning” (c), “Exclusively or mostly baby‐led weaning” (d + e) (see Table 1).

**Table 1:** Participant characteristics (*N* = 445).

Participant characteristics	*N* (%)
Gender	
Male	110 (24.7%)
Female	334 (75.1%)
Missing	1 (0.2%)
Age group	
18–24	8 (1.8%)
25–34	265 (59.6%)
35–44	163 (36.6%)
45–50	9 (2.0%)
Education	
Some high school or less	8 (1.8%)
High school diploma or equivalent (GED)	50 (11.2%)
Some college education	81 (18.2%)
Associate degree (AA) or vocational licence	25 (5.6%)
Bachelor's degree (BA, BS)	184 (41.3%)
Graduate or professional degree (MA, MSc, PhD, MD, JD)	94 (21.1%)
Prefer not to say	3 (0.7%)
Household income (average = £34,500, UK 2023^a^)	
< £25,000	31 (7.0%)
£25,000 to £49,999	138 (31.0%)
£50,000 to £74,999	151 (33.9%)
> £75,000	112 (25.2%)
Prefer not to say	13 (2.9%)
Marital status	
Single parent	21 (4.7%)
Co‐habiting	163 (36.6%)
Married	261 (58.7%)
Ethnicity	
Asian or Asian British	19 (4.3%)
Black or Black British	16 (3.6%)
White	403 (90.6%)
Any other groups	7 (1.6%)
Feeding method in the first 3 months	
Entirely or mostly breastfeeding	254 (57.1%)
Equally, entirely or mostly formula feeding	190 (42.7%)
Missing	1 (0.2%)
Complementary feeding approach in the first few months	
Exclusively or mostly spoon feeding	95 (21.3%)
A combination of spoon feeding and baby‐led weaning	156 (35.1%)
Exclusively or mostly baby‐led weaning	158 (35.5%)
Prefer not to say/missing	36 (8.1%)
Feeding routine	
I fed my baby whenever they cried, got fussy or seemed hungry.	284 (63.8%)
My baby was fed on a flexible schedule (e.g. every 3–4 h).	150 (33.7%)
My baby was fed on a rigid schedule (e.g. I woke them up to eat on time).	11 (2.5%)
Return to work before the baby was 6‐month old	
Yes	65 (14.6%)
No	378 (84.9%)
Prefer not to say	2 (0.4%)
Alexithymia status	
Yes (score above 60 on TAS‐20)	64 (14.4%)
No (score 0–60 on TAS‐20)	381 (85.6%)
Infant gender	
Male	236 (53.0%)
Female	208 (46.7%)
Prefer not to say	1 (0.2%)

^a^ Office for National Statistics, ONS https://www.ons.gov.uk/.

To test H1 and H2, correlation analyses were conducted between individual's alexithymia score, their RF practices, and their experienced family mealtime emotions. Due to the potential association between caregivers' alexithymia and their ability to identify and report their mealtime emotions, only Efficacy from MEM‐P was included in the corresponding analyses. It contains five items concerning mealtime experiences related to caregivers being prepared, comfortable, in control of their own emotions, and confident in dealing with any child distress. Next, hierarchical regression analyses were performed, with caregivers' gender, age, education background, feeding method in the first 3 months of infant age, complementary feeding approach, infant gender, and infant age controlled in the corresponding models.

To test H3, correlation analyses were applied to investigate the association between caregivers' general appetite traits, their sensitivity to internal satiation cues, and caregivers' RF practices. Then hierarchical regression was conducted with a variety of measures regarding individual's responsiveness to satiation entered. Hunot et al. (2016) demonstrated that SR is a highly heritable and stable eating trait. Substantial evidence reported that intuitive eating is stable over a 3‐week period (Tylka 2006; Tylka and Kroon Van Diest 2013; Tylka et al. 2024). Therefore, followed covariates in the regression, SR was added into Model 2, Reliance on Hunger and Satiety Cues was added into Model 3. RISE‐Q15 measures individual's capacity to rely on their satiation to stop eating which might vary by meals (Chawner et al. 2022), Model 4 added Physical Satisfaction, followed by Decreased Food Appeal. Same covariates as H1 were controlled in the corresponding model.

To test H4a and H4b, multivariate multiple regression analysis was conducted to assess the relationship between parent‐reported child appetitive traits and caregivers' RF practices. Confirmatory Factor Analysis (CFA) was performed to examine the latent variable Caregiver's Ability to Tune‐in to Own Satiation Cues (with Reliance on Hunger and Satiety Cues, and Physical Satisfaction). To assess goodness of model fit, chi square/degree of freedom (*χ* ^2^/df), the Comparison fit index (CFI ≥ 0.95), the Tucker‐Lewis fit index (TLI ≥ 0.95), Root mean square error of approximation (RMSEA < 0.08) and Standardised root mean square residual (SRMR < 0.08) were used holistically. With the identified CFA model, structural equation modelling (SEM) was conducted to explore the impact of caregivers' alexithymia on their awareness of infant hunger and satiety cues through reduced ability to “tune in” to their own appetite cues. Composite Reliability and Average Variance Extracted were calculated to assess the reliability and validity of the corresponding construct. Data were tidied and analysed via SPSS v29 and SPSS AMOS 29. Results were considered significant at *p* < 0.05.

#### 2.4.2 Qualitative Data

Responses to the open‐ended question were collated from Qualtrics and uploaded to Microsoft Excel for Relational Content Analysis (Busch et al. 2005; Krippendorff 2018), which explored the relationships among identified concepts in a text. Through Content Analysis, the study sought to identify key themes related to feeding timing, methods, and emotional aspects, and how these perceptions influenced their RF practices.

First, responses were read and then analysed to identify recurring themes and patterns. In the second stage, the units of meaning and the set of categories for coding were defined to reflect the core aspects of mealtime interactions. Next, a set of rules for coding included organising the units of meaning into the previously defined categories. Last, the researcher (SY) coded the data set then formulated themes, and summarised the qualitative data. Discrepancies were resolved through discussion with the other authors.

Based on participants' alexithymia score measured by TAS‐20, the first tertile and the third tertile were identified as two subgroups, which allowed the exploration of the qualitative data between caregivers who scored high on alexithymia, compared to those who scored low on alexithymia. Binary coding was applied to the data set. Participants scored 1 if their answers to the open‐ended question contained descriptors of positive affect; and 0 if their answers did not contain any descriptors of positive affect. The same coding approach was applied with descriptors of negative affect. Moreover, participants scored 1 if they indicated RF practices such as allowing their child to take control over the spoon, caregivers' observation of child appetitive cues, feelings generated from mealtimes; participants scored 0 if they simply described the foods offered during mealtimes, or their answers did not contain the previous elements and showed an externally oriented thinking style.

Chi‐square test of independence was applied to investigate if participants who scored high on alexithymia differed in reporting positive and/or negative affect, and mealtime interactions during feeding from those who scored low on alexithymia. Qualitative insights were used to contextualise and deepen the interpretation of the quantitative findings, emphasising how caregivers' emotional and relational dynamics during mealtimes influenced their RF behaviours.

### 2.5 Ethics Statement

Ethical approval for this study was granted by the University of Leeds, School of Psychology Ethics Committee (Reference: PSCETHS‐680). Informed consent was obtained from the participants of the study and their anonymised information could be published in this article. The relevant documents are available when requested by the journal.

## 3 Results

### 3.1 Participants

Overall, 445 eligible responses were received from caregivers (mean age: 33.5 ± 4.7 years, age range from 19 to 49 years). Participant characteristics are presented in Table 1. Most were white, female and married. Most (41.3%) had been educated to degree level; and most had above average household income (https://www.ons.gov.uk/) Infants were mostly breastfed during the first 3 months (57%), using both spoon feeding and baby led weaning was most common (70%), mean age of infants was 16.5 ± 6.3 months (range: 5–28 m), 53% of them were male. In our sample, 14.4% participants (*N* = 64) were identified as experiencing alexithymia according to the TAS‐20 cut‐off point.

### 3.2 Correlation Analyses

Individuals who scored high on Alexithymia were less likely to rely on internal satiation cues to guide their eating behaviours (*r* = −0.18, *p* < 0.001 for Physical Satisfaction from RISE‐Q15; *r* =−0.20, *p* < 0.001 for Reliance on Hunger and Satiety Cues from IES‐2), had high Decreased Food Appeal measured by RISE‐Q15 (*r* = 0.21, *p* < 0.001, see Supplementary Table 1); and low scores on awareness of infant hunger and satiety cues during mealtimes (*r* = −0.30, *p* < 0.001, see Supplementary Table 2). Moreover, associations were found between parent‐reported child appetitive traits and caregivers' RF practices. For example, child appetitive traits were associated with parental concerns about infant undereating or overeating and weight status (see Supplementary Table 3).

### 3.3 Hierarchical Regression Analyses

To test the first hypotheses, two hierarchical regressions were conducted separately to explore the relationships between caregiver alexithymia and their awareness of infant's hunger and satiety cues, and positive emotions during mealtimes. Caregivers' gender, age, education background, feeding method in the first 3 m, complementary feeding approach, infant gender, and infant age were controlled for in both regressions. After controlling for covariates, alexithymia significantly predicted caregivers' awareness of infant hunger and satiety cues, *R* ^2^ = 0.12, *F*(7, 399) = 7.53, *p* < 0.001 (see Supplementary Table 4). In the second regression model, caregivers' alexithymia remained predictive of their mealtime positive emotions after controlling for covariates, *R* ^2^ = 0.12, *F*(7, 399) = 7.49, *p* < 0.001 (see Supplementary Table 5).

Hierarchical regression was also used to test the second hypothesis, that RF practices will be associated with more positive experiences of mealtimes. In addition to the covariates included previously, caregivers' alexithymia score was added to Model 2. Three subscales from the IFQ were included to capture RF practices: Awareness of Infant's Hunger and Satiety Cues, Feeding Infant on a Schedule, and Using Food to Calm Infant's Fussiness. Awareness of infant's Hunger and Satiety Cues was associated with caregivers' positive mealtimes emotions (*b*_Awareness_ = 0.71, *p* < 0.001). Using Food to Calm Infant's Fussiness was inversely associated with positive mealtime emotions (*b*_Fussiness_ = −0.14, *p* = 0.019). After controlling for covariates and caregiver's alexithymia (Model 2 in the Table 2), caregiver's Awareness of Infant Hunger and Satiety Cues remained predictive of their positive mealtime emotions (*b*_Awareness_ = 0.56, *p* < 0.001) but Using Food to Calm was no longer significant in Model 2. Caregiver's Awareness of Infant Hunger and Satiety Cues, in addition to alexithymia, explained a significant proportion of variance in their positive mealtime emotions (*R* ^2^ = 0.19, *F*(8, 396) = 9.71, *p* < 0.001) (see Table 2).

**Table 2:** Hierarchical linear regression reporting predictors of caregivers' positive emotions during mealtimes.

	Model 1		Model 2	
Variable	*B* [SE]	*β*	*B* [SE]	*β*
Constant	2.69 [0.49]		4.17 [0.78]	
IFQ—Awareness of Infant Hunger and Satiety Cues	0.71 [0.09]	0.37***	0.56 [0.09]	0.29***
IFQ—Feeding Infant on a Schedule	−0.04 [0.10]	−0.02	−0.02 [0.10]	−0.01
IFQ—Using Food to Calm Infant's Fussiness	−0.14 [0.06]	−0.11*	−0.10 [0.06]	−0.08
Caregiver's gender			−0.19 [0.11]	−0.08
Caregiver's age			0.00 [0.01]	−0.01
Caregiver's education			0.03 [0.04]	0.04
Infant gender			0.10 [0.10]	0.04
Infant age			0.00 [0.01]	0.00
Feeding method in the first 3 m			0.10 [0.11]	0.05
Complementary feeding approach			0.07 [0.07]	0.05
Caregiver's alexithymia			−0.02 [0.01]	−0.24***
*R* ^2^	0.14		0.19	
*F*	23.48***		9.71***	
Δ*R* ^2^			0.06	
Δ*F*			4.02***	

*Note: N* = 408. SE = Standard Error.

* *p* < 0.05

** *p* < 0.01

*** *p* < 0.001.

Hierarchical regression was used to test the third hypothesis, that higher caregiver sensitivity to their own internal satiation cues, higher intuitive eating and interoceptive reasons for eating cessation are associated with higher sensitivity to infant hunger and satiety cues during mealtimes. Same covariates as hypothesis 1 were included. Overall, Model 5 accounted for approximately 11% of the variance in caregivers' Awareness of Infant Hunger and Satiety Cues, *F*(1, 396) = 5.40, *p *< 0.001. The addition of Decreased Food Appeal did not account for a significant increase in Model 5, Δ*F*(1, 396) = 1.06, *p* = 0.305, but caregivers' Satiety Responsiveness, intuitive eating, Physical Satisfaction and their gender were predictive of their Awareness of Infant's Hunger and Satiety Cues. In summary, mothers who are more tuning into their internal satiation cues and intuitive, who rely more on gastric fullness to guide their eating behaviours, are more likely to recognise their infant's hunger and satiety cues during mealtimes and respond accordingly (see Table 3).

**Table 3:** Hierarchical linear regression reporting predictors of caregivers' Awareness of Infant Hunger and Satiety Cues during mealtimes.

	Model 1		Model 2		Model 3		Model 4		Model 5	
Variable	*B* [SE]	*β*	*B* [SE]	*β*	*B* [SE]	*β*	*B* [SE]	*β*	*B* [SE]	*β*
Constant	3.78 [0.30]		3.87 [0.31]		3.03 [0.34]		2.86 [0.34]		2.90 [0.34]	
Caregiver's gender	0.10 [0.06]	0.09	0.12 [0.06]	0.10	0.15 [0.06]	0.12*	0.13 [0.06]	0.11*	0.13 [0.06]	0.11*
Caregiver's age	0.00 [0.01]	0.00	0.00 [0.01]	0.00	0.00 [0.01]	0.02	0.00 [0.01]	0.02	0.00 [0.01]	0.01
Caregiver's education	−0.02 [0.02]	−0.04	−0.02 [0.02]	−0.05	‐0.02 [0.02]	‐0.05	−0.02 [0.02]	−0.05	−0.02 [0.02]	−0.05
Infant gender	0.05 [0.06]	0.04	0.05 [0.06]	0.04	0.03 [0.06]	0.03	0.02 [0.05]	0.02	0.02 [0.05]	0.02
Infant age	0.00 [0.01]	0.04	0.00 [0.01]	0.04	0.00 [0.01]	0.03	0.00 [0.00]	0.03	0.00 [0.00]	0.03
Feeding method in the first 3 m	0.06 [0.06]	0.06	0.07 [0.06]	0.06	0.08 [0.06]	0.07	0.08 [0.06]	0.07	0.08 [0.06]	0.07
Complementary feeding approach	0.02 [0.04]	0.02	0.01 [0.04]	0.02	0.03 [0.04]	0.04	0.02 [0.04]	0.02	0.02 [0.04]	0.03
AEBQ – SR			−0.04 [0.04]	−0.05	−0.06 [0.04]	−0.09	−0.09 [0.04]	−0.13**	−0.08 [0.04]	−0.11*
IES2 – RHCS					0.20 [0.04]	0.26***	0.14 [0.04]	0.18***	0.13 [0.04]	0.17**
RISE‐Q15 – PS							0.12 [0.03]	0.24***	0.12 [0.03]	0.24***
RISE‐Q15 – DFA									−0.03 [0.03]	−0.05
*R* ^2^	0.00		0.00		0.06		0.11		0.11	
*F*	1.01		1.01		4.01***		5.83***		5.40***	
Δ*R* ^2^			0.00		0.06		0.05		0.00	
Δ*F*			1.02		27.45***		20.50***		1.06	

*Note: N* = 408. SE = Standard Error.

* *p* < 0.05

** *p* < 0.01

*** *p* < 0.001.

Multivariate multiple regression was used to examine the fourth hypotheses, that caregivers' RF practices measured by IFQ—Feeding Infant on a Schedule, and IFQ—Using Food to Calm Infant's Fussiness were associated with caregivers' eating traits, and with their child appetitive traits. Covariates were the same as H1. Results showed that CEBQ‐T measured child FR and SR, but not FF, were predictive of caregivers' using food to calm (see Supplementary Table 6). Feeding method in the first 3‐month of infancy was associated with caregivers' using food to calm (*b* = −0.42, *p* < 0.001). Complementary feeding approach in the first few months was related to caregivers' using food to calm, but the relationship was weak (*b* = 0.12, *p* = 0.037). No significant associations were found between caregivers' SR, intuitive eating and interoceptive reasons to stop eating and their Feeding Infant on a Schedule, or their Using Food to Calm Infant's Fussiness (data not shown). No relationship was found between child appetitive traits and caregiver's Feeding Infant on a Schedule (data not shown).

### 3.4 Structural Equation Modelling

**Figure 2:**
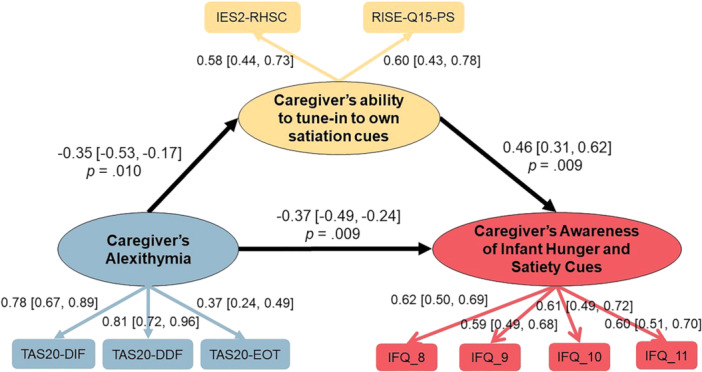
SEM model testing the structure of relationships between caregiver's alexithymia, ability to tune‐in to internal satiation cues and their awareness of infant hunger and satiety cues during mealtimes. Standardised estimates are reported with bias‐corrected bootstrapped confidence intervals.

To explore the association between caregivers' alexithymia and their awareness of infant's hunger and satiety cues through reduced sensitivity to their own appetite cues, CFA was performed to examine the latent variable “caregiver's ability to tune‐in to own satiation cues” which consisted Reliance on Hunger and Satiety Cues from IES‐2, and Physical Satisfaction from RISE‐Q15. SEM was conducted to examine the relationship between variables proposed in the model (see Figure 2).

SEM results demonstrated the standardised beta coefficients reported for the factor loadings onto latent variables and the relationships between each variable. All factor loadings onto latent variables were significant (*p* < 0.001). Overall model fit using the robust estimator was good (*χ* ^2^ (df = 24) = 54.15, *p* < 0.001, CFI = 0.96, TFI = 0.94, RMSEA = 0.05). However, the CR value fell below the threshold of 0.6 (CR = 0.52) (Bagozzi and Yi 1988), and the latent variable explained 35% of the variance (AVE = 0.35). Caregiver's alexithymia has an inverse association with their ability to tune in to their own internal satiation cues, as well as their Awareness of Infant Hunger and Satiety Cues during mealtimes (*r* = −0.35, *p* = 0.01; *r* = −0.37, *p* = 0.009 respectively). Positive relationship was observed between caregiver's ability to tune‐in to own satiation cues and their ability to recognise infant hunger and satiety cues (*r* = 0.46, *p* = 0.009).

### 3.5 Qualitative Data

Descriptions of a single, most recent mealtime interaction were analysed and content collated into frequently used themes and topics. From relational content analysis four themes were identified and are summarised below.

#### 3.5.1 Affect

The affective tone of the comments was generally positive, with descriptions such as “*happy*”, “*relaxed*”, “*pleasant/pleased*”, “*fun*”, “*calm*”, the caregiver‐infant dyad “*enjoyed*” the mealtime or their infant “*enjoyed*” the food, with “*happy”* being the most frequently mentioned descriptor. Negative affect was also captured. Some parents described feelings of “*frustration*” or “*pressure*”, arising from “*child being fussy*”, a lack of eating or food refusal, or child mealtime behaviours including “*screaming*”, “*throw foods*”, “*won't sit in the highchair*” or “*act up at times*”. Other negative descriptors included “*stress*” due to “*meal preparation*” or “*concerns of choking*”.

#### 3.5.2 Infant Appetite Cues

Participants reported a variety of behavioural and verbal signs indicating that their infant's interest or disinterest in eating. Infants expressed interest in eating with “*yummy*” or vocalisation, and “*done*” or scream for satiation. Some parents described that their child “*reached*” or “*picked up*” foods, “*leaned in*”, “*opened mouth*”, and “*woke up with cries*” to show hunger. On the contrary, children “*refused the food/spoon*”, “*threw/played with foods*”, “*closed mouth*” or “*waved hands*”, and “*tried to escape from the highchair*” when they were full. A small number of mixed behaviours signs were observed by participants. For example, a caregiver reported “*my child was still moaning so I offered more foods which was refused*”; whereas some children “*seemed more interested in playing despite clearly still wanting food, for example subsequently finishing the food*”. Moreover, participants' RF practices were captured through comments indicating parental awareness of their child's appetite cues, such as “*I feel like he knew he was full*”, “*my daughter feeds on demand*”, and “*I won't force him to eat if he isn't hungry or interested in the food.”*

#### 3.5.3 Feeding Methods

Spoon‐fed and baby‐led weaning were both observed in the sample. Some caregivers offered control to their child, encouraging them to feed themselves because they “*like the child to have control over what he eats*” or “*enjoy leaving her to eat at her own pace*”. They believe that this is “*completely developmental*”, allows their child to “*feel and play with foods”* and “*explore their meals*”. Some caregivers reported that they were “*spoon feeding initially then the child took over*”. In thinking of the use of spoons, a few caregivers stated that their child is self‐fed but “*if I offer to spoon feed, she'll eat more*” or “*I wanted her to eat more.*” In this case, the spoon is used as means to ensure more food is consumed, in addition to self‐feeding.

Participants mentioned they enjoyed “*sitting/eating as a family*” and they would “*share same food*” during mealtimes. The variety and nutritional value of foods were noted by parents, offering “*a selection of foods*” and that they would “*feel guilty*” if it was not nutritious enough or not home‐made. Participants reported that they actively encouraged their child to eat or try new foods (e.g. “*he didn't eat a lot of it, but he did try all the food so that pleased me*).

#### 3.5.4 Mealtime Environment and Interactions

Parental reflections on the feeding environment and mealtime interactions were well represented within the qualitative data. For example, participants mentioned they valued the time that they could “*spend together as a family*”, especially *after work;* that they enjoyed “*watching*” or “*observing*” their child during mealtimes. Different approaches were discussed to enhance verbal communication and connection during meals (“*chat about our day/things around*”, “*try to make him laugh*” and “*sing*”). Self‐reflections on positive interactions and parental intention to improve child engagement during mealtimes were observed, such as *“I find it really relaxing to take that time to focus on nothing else but my daughter*”. Notably, qualitative data revealed various feeding environment. Some caregivers intended to make mealtime interactive and fun “with nursery rhymes”, while some caregivers reported their experiences with TV (e.g. “*she was distracted by the TV which helps her eat more sometimes*”, “*we didn't interact much because we were watching TV*”). A number of caregivers illustrated the desire of distraction‐free mealtimes so their child can focus on the meal (e.g. “*I turned the TV off so there were no distractions for us both*”). Overall, caregivers expected and enjoyed good interactions during mealtimes, even if they were aware of “*the mess*” their infant would create through eating and the “*clean‐up*” afterwards.

The chi‐square test of independence revealed that there was no significant association between two groups of participants and their use of positive affect (*χ*²(1, *N* = 320) = 2.67, *p* = 0.103), or negative affect (*χ*²(1, *N* = 320) = 1.95, *p* = 0.163) in describing their most recent mealtime experiences, or their self‐reported mealtime interactions regarding caregivers being responsive during feeding (*χ*²(1, *N* = 320) = 1.65, *p* = 0.199).

## 4 Discussion

This study investigated the relationships between caregiver attributes including alexithymia, sensitivity towards own satiation cues, and child appetitive traits with caregivers' mealtime emotions and RF practices. Our first hypothesis was supported, indicating higher caregiver alexithymia scores were associated with fewer RF practices and less positive mealtime experiences. Partial support for the second hypothesis indicated that greater awareness of infant hunger and satiety cues (but not other aspects of RF practices) was associated with more positive mealtime experiences. The third hypothesis was again supported with results indicating caregiver satiety responsiveness, intuitive eating and physical satisfaction, all associated with their awareness of infant hunger and satiety cues during mealtimes. Finally, partial support was found for hypothesis four with child appetitive traits (higher food responsiveness and higher satiety responsiveness) positively associated with caregiver using food to calm their infant but not feeding on a schedule. These findings supported the proposal that caregivers who were more responsive during mealtimes, were more likely to experience positive emotions during meals. However, caregivers who scored high on alexithymia reported lower levels of being responsive in feeding, in addition to fewer positive mealtime emotions, compared to those who score low on alexithymia.

SEM results suggested that alexithymia was associated with reduced caregivers' awareness of infant's hunger and satiety cues via reduced sensitivity to caregivers' own appetite cues. Caregivers who scored high on alexithymia were less likely to recognise and attend to their infant's appetite cues during feeding; meanwhile, this inverse association was related to caregivers' reduced ability to recognise and respond (“tune in”) to their own satiation cues. This finding supports the proposal that alexithymia is a general deficit of interoception, including non‐affective interoceptive states (Brewer et al. 2016).

The ability to recognise both affective and non‐affective cues is particularly important serving as the basis to respond to another's needs (Brewer et al. 2016). An impaired ability to “decode” infant cues may relate to overall RF practices. For example, parents may apply nonresponsive feeding practices such as coercive strategies if they are concerned that their child is not eating enough (Birch et al. 1991).

The present SEM results also revealed that the reliability of the proposed construct, named “Caregivers' Ability to Tune‐in to Their Own Internal Satiation Cues” could be improved. This may be due to Reliance on Hunger and Satiety Cues from the intuitive eating measurement and Physical Satisfaction from RISE‐Q15 were measuring different aspects of individual's sensitivity to their interoception. Additionally, Reliance on Hunger and Satiety Cues is a trait that is stable for over 3‐week period (Tylka 2006; Tylka and Kroon Van Diest 2013; Tylka et al. 2024). Whereas Physical Satisfaction from the RISE‐Q15 reflects an individual's response to satiation during a typical eating episode and a tendency towards eating cessation for reasons of gastric fullness (e.g., “I stop eating at a typical dinner meal at home because my stomach is full”). Moreover, the results relied on participants' self‐reported answers. The reliability of the proposed construct could be improved by including more objective measures, for example, laboratory assessments of appetite responses.

Caregivers of infants with high food responsiveness (FR) and high satiety responsiveness (SR) were more likely to use food to soothe compared to those whose children scored lower on FR and SR, partially supporting findings from previous observational and intervention studies that eating traits indicating an avid appetite were associated with less responsive maternal feeding practices (Daniels et al. 2014; Morrison et al. 2013; Rodgers et al. 2013). The bidirectional association between parental feeding practices and child eating behaviours revealed that parental feeding practices were not simply a predictor or a consequence of certain child appetitive traits (Costa and Oliveira 2023). For example, high infant FR was associated with increased parental use of food to control children's behaviours (Edwards et al. 2024; Kininmonth et al. 2023). Researchers from the longitudinal Gemini cohort study reported that nonresponsive feeding practices including coercive feeding or parental over‐control may undermine their child's capacity to identify their internal hunger and satiety cues (Kininmonth et al. 2023). Our findings indicated that less desirable feeding practices were used in a home setting as a response to infants displaying higher levels of FR and SR.

Existing literature demonstrates that mothers need to assign intent to their child's signals and to interpret them in a timely and accurate way so that their response is both sensitive and appropriate (Farrow and Blissett 2014; Meins 1999). The importance of interactions in shared family mealtimes, involving verbal language and body language has been reported by Van der Heijden and Wiggins (2025), which is associated with what, when and how much caregivers and their children eat. Caregivers who “tune‐in” to their child expressing needs, satisfy their child and provide healthy interaction, rather than merely focusing on the act of feeding.

Participants with higher alexithymia scores did not differ from those with lower alexithymia scores in frequency of reported positive or negative affect or indicators of responsive feeding when describing their mealtime experiences. This may be because although participants with higher alexithymia scores may have differed in their general ability to identify and express their emotions and feelings, the open‐ended question specifically asked “how did you feel” as a clear prompt. The binary coding only indicated whether or not they were aware of their affect during the given feeding occasion, rather than to what extent they were good at identifying and describing their emotions and feelings (i.e. alexithymia). However, multiple approaches to enhance mealtime interactions were recorded in our qualitative data, such as less environmental distraction, verbal communication during feeding, and increasing child involvement in food preparation or the family meal. This supported the finding from an earlier observational study that having mealtime structure might be an effective strategy to promote parental role modelling healthy eating and reduce child fussy eating (Powell et al. 2017). The acknowledgement of the bidirectional interaction between caregiver‐infant dyad during feeding could contribute to the development of tailored interventions and healthier feeding guidance (Moore et al. 2007; Webber et al. 2010). These findings have highlighted the importance of caregiver's capacity to understand the intention underlying infant appetite cues so they could offer prompt, contingent and appropriate response to their infant. In addition, the results demonstrated the association between caregiver's ability to tune in to own satiation cues and their recognition of infant hunger and satiety cues, specifically highlighting the negative impact from caregiver's alexithymia on RF practices. The present study suggested that strategies to enhance caregiver's awareness and responsiveness to infant affect and appetite may improve RF practices and healthy mealtime interactions.

### 4.1 Strengths and Limitations

One strength of this study was its size, and so is the first study appropriately powered to explore the associations between caregiver's RF practices, caregiver's psychological attributes including tune‐in to internal satiation cues, alexithymia, and mealtime emotions. Another strength is the inclusion of an open‐ended question about mealtimes, which permitted additional insight beyond the quantitative measures regarding the tone and content of feeding experiences through the freedom to report any aspect of mealtime interactions. This then added context to the quantitative measures of RF. The inclusion of TAS‐20 enabled further analyses of alexithymia within the sub‐group. Moreover, good internal association observed between caregivers' SR, intuitive eating, and satiation response provided evidence to support the identified aggregated construct which represents an individual's “sensitivity to internal satiation cues” in prior research (Chawner et al. 2022). However, the study is limited by its single “snapshot” of a mealtime and a lack of representativeness of the sample. Additionally, with reliance on self‐report, combined with time constraints and large sample size, it was not possible to ask participants to film their feeding practices at home to support their response to the open‐ended question. This prevented a validation on self‐reported measures of responsiveness to infant appetite cues with observational evidence (Bergmeier et al. 2015), which might introduce recall‐bias in describing the mealtime interactions. Another limitation lies in the nature of the online survey where researchers rely on participants being genuine and honest. Finally, caregivers' autism spectrum disorder, depression, or other mental health conditions that are known to be more prevalent in individuals with alexithymia were not assessed in the present study.

## 5 Conclusion

Caregivers with high scores on alexithymia reported fewer RF practices and experienced less positive mealtime emotions, compared to those with low scores on alexithymia. Positive mealtime emotions were observed within caregivers scoring high on RF practices. Mothers who were “in‐tune” with their own internal satiation cues, were more likely to recognise their infant hunger and satiety cues during feeding, than those who were less responsive to internal cues. Overall, caregivers were more likely to use food to calm in response to their child displaying high SR and FR. Most importantly, alexithymia was associated with caregivers' awareness of their infant's hunger and satiety cues during feeding through reduced awareness of their own appetite cues. Future studies could investigate the barriers for caregivers with alexithymia to apply RF practices, and how caregivers could be supported to help children to develop healthy eating behaviours (Saltzman et al. 2018).

## Author Contributions

**Shihui Yu:** writing – review and editing, writing – original draft, validation, project administration, methodology, investigation, formal analysis, data curation, conceptualisation. **Alison Fildes:** writing – review and editing, writing – original draft, supervision, methodology. **Pam Birtill:** writing – review and editing, writing – original draft, validation, supervision, methodology. **Tang Tang:** writing – review and editing, writing – original draft, supervision, methodology, conceptualisation. **Marion M. Hetherington:** writing – review and editing, writing – original draft, validation, supervision, methodology, investigation, formal analysis, conceptualisation.

## Conflicts of Interest

The authors declare no conflicts of interest.

## Supporting information


**Supporting Table 1:** Correlation analyses between individual's appetite traits and Alexithymia status. **Supporting Table 2:** Correlation analyses between individual's Alexithymia status, RF practices and positive mealtime emotions. **Supporting Table 3:** Correlation analyses between caregiver's RF practices and caregiver reported child appetite traits. **Supporting Table 4:** Hierarchical Linear Regression reporting predictors of caregiver's Awareness of Infant Hunger and Satiety Cues. **Supporting Table 5:** Hierarchical Linear Regression reporting predictors of caregiver's positive emotions during mealtimes. **Supporting Table 6:** Multivariate regression reporting predictors of caregiver's Using Food to Calm Infant's Fussiness.

## Data Availability

The data that support the findings of this study are available on request from the corresponding author. The data are not publicly available due to privacy or ethical restrictions.
