# Supplementary material for: Tuning Into Affect and Appetite in Caregivers, and Its Association With Recognising and Responding to Infant Appetite Cues
Source: Matern Child Nutr. 2025 Aug 31;22(1):e70099. doi: 10.1111/mcn.70099 (PMC12893506; doi:10.1111/mcn.70099)
Supplement: Supplementary file 1 — Supporting Table 1: Correlation analyses between individual's appetite traits and Alexithymia status. Supporting Table 2: Correlation analyses between individual's Alexithymia status, RF practices and positive mealtime emotions. Supporting Table 3: Correlation analyses between caregiver's RF practices and caregiver reported child appetite traits. Supporting Table 4: Hierarchical Linear Regression reporting predictors of caregiver's Awareness of Infant Hunger and Satiety Cues. Supporting Table 5: Hierarchical Linear Regression reporting predictors of caregiver's positive emotions during mealtimes. Supporting Table 6: Multivariate regression reporting predictors of caregiver's Using Food to Calm Infant's Fussiness. [file MCN-22-e70099-s001.docx]

# Supplementary materials

|  | 1. AEBQ-Satiety Responsiveness | 2. IES-2-Reliance on Hunger and Satiety Cues | 3. RISEQ-15- Physical Satisfaction | 4. RISEQ-15-Decreased Food Appeal | 5. TAS-20 sum |
| --- | --- | --- | --- | --- | --- |
| 1 | - | .122** | .255*** | .344*** | -.031 |
| 2 |  | - | .349*** | -.057 | -.200*** |
| 3 |  |  | - | .138** | -.177*** |
| 4 |  |  |  | - | .209*** |

Supplementary table 1. Correlation analyses between individual’s appetite traits and Alexithymia status. ***p* < 0.01, ****p* < 0.001. N = 445.

|  | MEM-P-Efficacy | 1 | 2 | 3 | 4 | 5 | 6 | 7 |
| --- | --- | --- | --- | --- | --- | --- | --- | --- |
| TAS20-sum | -.357*** | -.301*** | .110* | .120* | .080 | .051 | .075 | -.043 |
| MEM-P-Efficacy | - | .341*** | -.245*** | -.180*** | -.093* | -.040 | -.095* | .083 |

Supplementary table 2. Correlation analyses between individual’s Alexithymia status, RF practices and positive mealtime emotions. 1: IFQ-Awareness of Infant’s Hunger and Satiety Cues; 2: IFQ-Concern about Infant Undereating or Becoming Underweight; 3: IFQ-Concern About Infant Overeating or Becoming Overweight; 4: IFQ-Concern About Infant's Hunger; 5: IFQ-Feeding Infant on a Schedule; 6: IFQ-Using Food to Calm Infant's Fussiness; 7: IFQ-Social Interaction with the Infant During Feeding. **p* <0.05, ***p* < 0.01, ****p* < 0.001. N = 445.

|  | 1 | 2 | 3 | 4 | 5 | 6 | 7 |
| --- | --- | --- | --- | --- | --- | --- | --- |
| CEBQ-T-Food Responsiveness | -.105* | -.291*** | .319*** | .129** | .036 | .175*** | -.038 |
| CEBQ-T-Satiety Responsiveness | -.040 | .422*** | -.208*** | -.091 | -.070 | .007 | .038 |
| CEBQ-T-Food Fussiness | -.224*** | .387*** | -.058 | -.037 | -.045 | .027 | .012 |
| CEBQ-T-Slowness in Eating | -.082 | .297*** | -.085 | .003 | .020 | -.049 | .036 |
| CEBQ-T-Enjoyment of Food | .250*** | -.405*** | .099* | .026 | .079 | .029 | .024 |

Supplementary table 3. Correlation analyses between caregiver’s RF practices and caregiver reported child appetite traits. 1: IFQ-Awareness of Infant’s Hunger And Satiety cues; 2: IFQ-Concern about Infant Undereating or Becoming Underweight; 3: IFQ-Concern About Infant Overeating or Becoming Overweight; 4: IFQ-Concern About Infant's Hunger; 5: IFQ-Feeding Infant on a Schedule; 6: IFQ-Using Food to Calm Infant's Fussiness; 7: IFQ-Social Interaction with the Infant During Feeding. **p* <0.05, ***p* < 0.01, ****p* < 0.001. N = 445.

|  | Model 1 | | Model 2 | |
| --- | --- | --- | --- | --- |
| Variable | B [SE] | β | B [SE] | β |
| Constant | 4.97 [0.12] |  | 4.82 [0.32] |  |
| Caregiver’s alexithymia | -0.02 [0.00] | -0.33*** | -0.02 [0.00] | -0.35*** |
| Caregiver’s gender |  |  | 0.10 [0.06] | 0.08 |
| Caregiver’s age |  |  | -0.00 [0.01] | -0.03 |
| Caregiver’s education |  |  | -0.04 [0.02] | -0.09 |
| Infant gender |  |  | 0.04 [0.05] | 0.03 |
| Infant age |  |  | 0.01 [0.00] | 0.05 |
| Feeding method in the first 3m |  |  | 0.06 [0.06] | 0.05 |
| Complementary feeding approach |  |  | 0.04 [0.04] | 0.06 |
|  |  |  |  |  |
| R^2^ | 0.10 | | 0.12 | |
| F | 48.40*** | | 7.65*** | |
| ΔR^2^ |  | | 0.03 | |
| ΔF |  | | 1.74 | |

Note: N = 408. SE = Standard Error

**p*<.05; ***p*<.01; ****p*<.001

Supplementary table 4. Hierarchical Linear Regression reporting predictors of caregiver’s Awareness of Infant Hunger and Satiety Cues.

|  | Model 1 | | Model 2 | |
| --- | --- | --- | --- | --- |
| Variable | B [SE] | β | B [SE] | β |
| Constant | 6.71 [0.23] |  | 6.48 [0.61] |  |
| Caregiver’s alexithymia | -0.03 [0.01] | -0.35*** | -0.04 [0.01] | -0.35*** |
| Caregiver’s gender |  |  | -0.14 [0.11] | -0.06 |
| Caregiver’s age |  |  | -0.01 [0.01] | -0.02 |
| Caregiver’s education |  |  | 0.01 [0.04] | 0.01 |
| Infant gender |  |  | 0.12 [0.10] | 0.06 |
| Infant age |  |  | 0.00 [0.01] | 0.02 |
| Feeding method in the first 3m |  |  | 0.18 [0.11] | 0.08 |
| Complementary feeding approach |  |  | 0.08 [0.07] | 0.05 |
|  |  |  |  |  |
| R^2^ | 0.12 | | 0.12 | |
| F | 55.23*** | | 7.72*** | |
| ΔR^2^ |  | | 0.01 | |
| ΔF |  | | 0.94 | |

Note: N = 408. SE = Standard Error

**p*<.05; ***p*<.01; ****p*<.001

Supplementary table 5. Hierarchical Linear Regression reporting predictors of caregiver’s positive emotions during mealtimes.

| Variables | IFQ – Using Food to Calm Infant’s Fussiness | |
| --- | --- | --- |
|  | β (SE) | *p* |
| Caregiver’s gender | 0.10 (0.09) | .29 |
| Caregiver’s age | 0.01 (0.01) | .47 |
| Caregiver’s education | 0.02 (0.03) | .60 |
| Infant’s gender | -0.07 (0.08) | .39 |
| Infant’s age | -0.01 (0.01) | .17 |
| Feeding method in the first 3m | -0.41 (0.09) | < .001 |
| Complementary feeding approach | 0.12 (0.06) | .037 |
| CEBQ – Food Responsiveness | 0.33 (0.06) | < .001 |
| CEBQ – Satiety Responsiveness | 0.26 (0.07) | < .001 |
| CEBQ – Food Fussiness | -0.01 (0.05) | .84 |

Note: N = 408. SE = Standard Error

Supplementary table 6. Multivariate regression reporting predictors of caregiver’s Using Food to Calm Infant’s Fussiness.
